# 4369 Reprogramming of vascular smooth muscle cells to multipotent progenitor cells contributes to progression of atherosclerosis

**DOI:** 10.1017/cts.2020.315

**Published:** 2020-07-29

**Authors:** Allison Milfred Dubner, Sizhao Lu, Austin Jolly, Keith Strand, Marie Mutryn, Rebecca Tucker, Karen Moulton, Raphael A. Nemenoff, Mary C.M. Weiser-Evans

**Affiliations:** 1 University of Colorado at Denver

## Abstract

OBJECTIVES/GOALS: Our lab previously identified a population of vascular smooth muscle (SMC)-derived progenitor cells (AdvSca1-SM) which expand robustly in response to disease and can differentiate into multiple cell types. We now aim to define the role of these AdvSca1-SM cells in atherosclerotic plaque progression. METHODS/STUDY POPULATION: Goal one uses SMC lineage tracing mice and a model of atherosclerosis to track reprogramming of SMCs to AdvSca1-SM cells in the setting of disease. Arteries are analyzed using flow cytometry and immunofluorescence to quantify changes in number of mature SMCs and AdvSca1-SM cells. Goal two uses AdvSca1-SM lineage tracing mice with high cholesterol-induced atherosclerosis and plaque neovascularization. Arteries are analyzed to quantify expansion of AdvSca1-SM cells, subsequent re-differentiation into mature SMC, endothelial cells, or macrophages, and contribution to plaque neovascularization. Mechanistic findings from both goals are being investigated in diseased human coronary arteries. RESULTS/ANTICIPATED RESULTS: Flow cytometry from SMC lineage tracing mice revealed a 7- to 13-fold expansion of AdvSca1-SM cells in carotid arteries (p<0.001) and aortas (p = 0.03) after 6 weeks of western diet; no differences in macrophage numbers were observed. Additional SMC and AdvSca1-SM cell lineage tracing mice are on atherogenic diets to assess early and advanced atherosclerosis. We predict that AdvSca1-SM cells will contribute to macrophage accumulation as well as plaque neovascularization in the setting of severe atherosclerosis. Translational relevance of mechanisms driving SMC reprogramming and AdvSca1-SM cell contribution to plaque progression are being applied to studies of diseased human coronary arteries. DISCUSSION/SIGNIFICANCE OF IMPACT: Our data suggest a role for AdvSca1-SM cells in atherosclerosis. Ongoing work will clarify the mechanisms driving plaque-associated AdvSca1-SM expansion and define the ultimate fates of these cells. *In vivo* modulation of this process could provide the basis for future anti-atherosclerotic therapies. CONFLICT OF INTEREST DESCRIPTION: AD - CCTSI TOTTS TL1TR002533; SL - 18POST34030397 from the American Heart Association; AJ – no conflicts; KS - 1F31HL147393 from the National Heart, Lung, and Blood Institute, NIH; MM – no conflicts; RT – no conflicts; KSM – no conflicts; RAN - R01CA236222 from the National Cancer Institute, NIH, and 2018-03 from the Lungevity Foundation; and MCMW-E - R01 HL121877 from the National Heart, Lung, and Blood Institute, NIH, and 25A8679 from the Chernowitz Foundation.

